# Longitudinal Change in the Relationship between Fundamental Motor Skills and Perceived Competence: Kindergarten to Grade 2

**DOI:** 10.3390/sports5030059

**Published:** 2017-08-10

**Authors:** Jeff R. Crane, John T. Foley, Patti-Jean Naylor, Viviene A. Temple

**Affiliations:** 1 School of Exercise Science, Physical & Health Education, University of Victoria; Victoria, BC V8P 5C2, Canada; pjnaylor@uvic.ca (P.-J.N.); vtemple@uvic.ca (V.A.T.); 2 Physical Education Department, State University of New York College at Cortland, Cortland, NY 13045, USA; john.foley@cortland.edu

**Keywords:** motor skills, motor competence, physical literacy, perceptions of competence, children, longitudinal, early childhood, middle childhood

## Abstract

As children transition from early to middle childhood, the relationship between motor skill proficiency and perceptions of physical competence should strengthen as skills improve and inflated early childhood perceptions decrease. This study examined change in motor skills and perceptions of physical competence and the relationship between those variables from kindergarten to grade 2. Participants were 250 boys and girls (Mean age = 5 years 8 months in kindergarten). Motor skills were assessed using the Test of Gross Motor Development-2 and perceptions were assessed using a pictorial scale of perceived competence. Mixed-design analyses of variance revealed there was a significant increase in object-control skills and perceptions from kindergarten to grade 2, but no change in locomotor skills. In kindergarten, linear regression showed that locomotor skills and object-control skills explained 10% and 9% of the variance, respectively, in perceived competence for girls, and 7% and 11%, respectively, for boys. In grade 2, locomotor skills predicted 11% and object-control skills predicted 19% of the variance in perceptions of physical competence, but only among the boys. Furthermore, the relationship between motor skills and perceptions of physical competence strengthened for boys only from early to middle childhood. However, it seems that forces other than motor skill proficiency influenced girls’ perceptions of their abilities in grade 2.

## 1. Introduction

Lower perceptions of physical or sport competence are associated with dropout from organized sport among children and youth [1] and avoidance of physical education [2], whereas higher perceptions of physical competence are consistently associated with greater participation in physical activity among children and youth [3,4,5,6]. With low levels of physical activity worldwide, it is important to understand the development of self-perceptions and motor skills in children and youth so participation can be enhanced.

As children transition from early to middle childhood, hypothetically two processes should strengthen the relationship between motor skill proficiency and perceptions of physical competence. First, motor skills generally improve during childhood [7,8,9,10,11], and second, perceptions of physical competence generally decrease as children develop cognitively [5,12,13,14]. Further, developmental theorists note that as children age and become more exposed to additional factors that influence their perceptions, they rely less on the feedback from significant others (e.g., parents and caretakers), and more on that from other sources (e.g., peers) [15]. Overall, motor skills are less developed in early childhood than in middle childhood [9]; however, children’s physical competence beliefs tend to be higher and less accurate in early childhood [13,15].

To date, the relationship between fundamental motor skill proficiency and perceived physical competence in early childhood is unclear. Five studies have examined this relationship using identical tools [1,16,17,18,19], specifically, the Test of Gross Motor Development-2 (TGMD-2; Ulrich, 2000) to assess motor proficiency and the Pictorial Scale of Perceived Competence and Acceptance for Young Children [20] to assess perceptions of physical competence. Three of the studies found significant positive relationships between either locomotor skill and perceptions and object control skills and perceptions, but Crane et al. [2] found that the relationship was only significant for boys. Contrastingly, two other studies did not find significant relationships between motor skill proficiency and perceptions of physical competence [16,19]. It is possible that the relationship in the Goodway and Rudisill [16] study differed because the children had extremely low motor skill scores. In slightly older children (8 years of age), Yu and colleagues [21] examined this relationship among children with developmental coordination disorder (DCD) and typically developing children. These authors reported that children with DCD had lower perceptions of their physical abilities and displayed lower motor proficiency levels than their typically developing peers. In addition, they found that physical coordination was a predictor of object control skills [21].

As they age, children’s expanding cognitive abilities enable greater awareness of their own competence and performances [22] and allow them to: compare their performances to their peers’ performances, analyze the reasons for their successes and failures, and internalize feedback [13,23,24], resulting in less inflated perceptions. As children mature, those with less proficient skills will more likely have less favourable physical competence perceptions, and those with well-developed motor skills more favourable [14], which can significantly influence physical activity and sport participation patterns. Although no studies have tracked the relationship between fundamental motor skills and perceived physical competence from early to middle childhood longitudinally, Spessato and colleagues [19] assessed this relationship in a cross-section of children at different ages. As might be expected developmentally, Spessato et al. found that the relationship was not significant among the 4- and 5-year-old children, but it was significant among the 6-year-old children.

Sex-based differences in fundamental motor skill proficiency as well as in the relationship between motor skills and perceptions have also been identified. Results of a recent systematic review and meta-analysis of the correlates of gross motor competence revealed that sex was a correlate of gross motor competence in more than 40 studies worldwide [25]. Boys have consistently shown higher object control skills and a stronger relationship between object control skills and perceptions of competence, compared to girls [10,12,17,18,26,27]. The evidence is less clear for locomotor skills. Several studies demonstrated that girls have better locomotor skill proficiency [10,12,18], while other studies found no differences [28]. For perceptions of physical competence, LeGear and colleagues [18] found that 5-year-old girls and boys had high perceptions of their physical competence, but that girls’ perceptions were significantly higher than boys’. On the other hand, Robinson [17] found that 4-year-old boys had higher levels than girls, and Goodway et al. [16] found no sex-based differences among 3–4-year-olds. However, current models such that presented by as Stodden et al. [15] have not included sex differences. Part of the impetus for this study was to test aspects of Stodden and colleagues’ [15] developmental model published in Quest. Stodden et al. hypothesized that in both early and middle childhood, perceptions of competence would predict motor skill proficiency, but for slightly different reasons. In early childhood, positive perceptions may help in the development of motor skills because the children do not really differentiate between the effort they put into their activity and the outcomes (i.e., success or failure), while in middle-childhood, improvements in motor skill proficiency coupled with positive perceptions of their ability should encourage children to continue to practice and refine their skills, which in turn leads to more positive perceptions. However, Stodden et al. [15] did not include gender in their model.

There is a need to further our understanding of the developmental trajectory of perceptions of physical competence and the relationship between perceptions and motor proficiency, especially from early to mid-childhood and between boys and girls. A longitudinal design was used to examine the relationship between fundamental motor skill proficiency and perceptions of physical competence from early childhood to the beginning of middle childhood. Focusing on children’s transition from kindergarten to grade 2, four hypotheses were tested: (1) that motor skill proficiency would increase, (2) that perceptions of physical competence would decrease, (3) that sex-based differences would be evident for motor skills and perceptions of physical competence, and (4) that the relationship between motor competence and perceived competence would strengthen.

## 2. Materials and Methods

### 2.1. Participants

Children were eligible to participate if they were attending one of eight consenting elementary schools in one school district in British Columbia, Canada. Census data from Statistics Canada indicates that, as of 2014, British Columbia families have a median income of approximately 10% higher than the national median [18]. In the school district used in the present study, rates of vulnerability (as measured by the Early Development Index) are lower than, or equivalent to other British Columbia school districts [13,19]. Two cohorts of children were examined. In kindergarten, participants for cohort one were recruited during the 2010–2011 school year (wave 1) and cohort two during the 2011–2012 school year (wave 2). These kindergarten cohorts were tracked to grade 2 using data collected between October and May 2012–2013 and 2013–2014. Children were included in the longitudinal sample if they had complete motor skills and perceptions of competence data in both kindergarten and grade 2. The University of Victoria Human Research Ethics Board and the School District approved this study. Parents provided consent and children provided assent.

### 2.2. Materials

Fundamental motor skills (six locomotor skills: run, jump, hop, slide, gallop, and leap; and six object control skills: throw, roll, kick, strike, catch, and dribble) were assessed using the TGMD-2 [9]—a criterion and norm-referenced test with established test-retest reliability and evidence of content, construct, and criterion validity [10]. Additionally, body mass index (BMI) was measured as a potential confounder.

*The Pictorial Scale of Perceived Competence and Acceptance for Young Children*—preschool and kindergarten [29] and *The Pictorial Scale of Perceived Competence and Acceptance for Young Children*—first and second grade [30] were used to assess perceptions of physical competence. Each scale consists of 24 items subdivided into four subscales (six statements each): Cognitive Competence, Physical Competence, Peer Acceptance, and Maternal Acceptance. Only the perception of physical competence subscale was used for this study. Both versions of the test contain the same number of questions, as well as the same subscales and script to be read; however, two skills were changed to be more age appropriate. For the physical domain, two questions (bouncing a ball and jumping rope) were added by Harter and Pike [20] to the grade 1 and 2 version of the survey, while tying one’s shoes and hopping on one foot were omitted. The surveys have acceptable reliability and validity for use with kindergarten and grade 2 children [20].

### 2.3. Procedures

A team of 10 trained research assistants collected these data. The TGMD-2 was administered during scheduled physical education classes in accordance with the testing procedure outlined in the Examiner’s Manual [9]. Each class was divided into four small groups prior to entering the gymnasium, with each group consisting of 3–5 children. Each consented child was digitally recorded performing the skills at their station twice before moving onto the next station. After all skills were recorded, trained research assistants measured the height and weight of each participant to determine BMI. Due to scheduling and time constraints, data were collected over multiple visits to each school. The Pictorial Scale of Perceived Competence and Acceptance for Young Children [20] was administered by the research assistants individually with each child in a quiet area.

### 2.4. Data Treatment and Analyses

The principal investigator scored the behavioural components of each of the 12 skills dichotomously using digital video. The number of components completed correctly for each subtest (locomotor and object control skills) was summed to provide a raw score (range 0–48). The items of both versions of The Pictorial Scale of Perceived Competence and Social Acceptance for Young Children assessing physical competence were scored on a scale of 1–4 for each item. Scores from the physical competence subscale questions were summed (six items total) to provide a raw score out of 24 (range 6–24). Descriptive statistics were then computed for locomotor skills, object control skills, and perceptions of physical competence in kindergarten and grade 2. Specifically, means and standard deviations, as well as percent of maximum possible score (POMP) [31] were calculated. POMP was calculated using the following equation: (Observed score − minimum possible/maximum possible − minimum possible) × 100.

To test whether motor skills improved (Hypothesis 1) and perceptions decreased (Hypothesis 2), we performed a mixed-design analysis of variance to examine the change in perceived competence, locomotor and object control skills over time using sex as the between-subject factor and grade level as the within-subjects factor. Further, paired-sample *t*-tests were conducted to examine change or stability in each of the 12 skills. Pearson product-moment correlation coefficients were computed to test the relationships between locomotor skills and perceptions of physical competence and between object control skills and perceptions of physical competence for boys and girls in kindergarten and in grade 2 (Hypotheses 3 and 4). Further, a series of linear regression analyses were conducted to predict perceptions of physical competence (as the outcome variable) from locomotor and object control skills (predictor variables) in kindergarten and grade 2 for boys and girls separately. All statistical analyses were conducted using IBM SPSS (Version 23.0, IBM Corp., Armonk, NY, USA) for Windows [32], and alpha value for rejecting the null hypothesis was set at <0.05 [33].

## 3. Results

Two hundred and fifty children (of 780 measured) had complete motor skills and perceptions of competence data in both kindergarten and grade 2 and were included in the longitudinal analysis. The mean age for children in kindergarten and grade 2 was 5.8 ± 0.3 years and 7.7 ± 0.4 years, respectively. Descriptive statistics grouped by sex for locomotor skills, object control skills, and perceptions of physical competence are reported in Table 1.

The raw and POMP motor skill scores indicated that the children’s skills in kindergarten were in the middle of the range of possible scores, but had increased to approximately 57% to 67% of the maximum possible by grade 2. Perceived physical competence scores were high in both grades.

The mixed analyses of variance revealed a significant improvement in object control skill raw scores from kindergarten to grade 2, Wilk’s Lambda = 0.873, *F*(1, 248) = 36.129, *p* = < 0.001, ηp2 = 0.13 as well as a significant effect for sex *F*(1, 248) = 29.992, *p* < 0.001, ηp2 = 0.11. Boys had significantly higher object control skills compared to girls in both kindergarten and grade 2 (see Table 1). There was no overall improvement in locomotor scores from kindergarten to grade 2; Wilk’s Lambda = 0.994, *F*(1, 248) = 1.611, *p* = 0.206, ηp2 = 0.006. However, there was a significant effect for sex, revealing that girls had significantly higher locomotor skills compared to boys in both kindergarten and grade 2 *F*(1, 248) = 8.806, *p* = 0.003, ηp2 = 0.04. There was also a significant increase overall in perceived physical competence from kindergarten to grade 2, as evidenced by a Wilk’s Lambda of 0.983, *F*(1, 248) = 4.257, *p* < 0.040, ηp2 = 0.02. In addition, there was a significant effect of gender on perceived physical competence *F*(1, 248) = 11.369, *p* = 0.001, ηp2 = 0.044, revealing that girls had higher perceptions of physical competence than boys in both kindergarten and grade 2.

Change or stability of each of the 12 TGMD-2 skills is presented in Table 2. Across both the boys and the girls, there was significant improvement in seven out of the 12 skills (run, hop, leap, slide, strike, dribble, and catch) from kindergarten to grade 2.

Sex-based differences were evident, with catching and rolling improving among boys and not girls, and gallop, leap, and jumping improving among girls and not boys.

In kindergarten, perceived physical competence and both locomotor skills and object control skills were significantly related for both girls and for boys (see Table 3). In grade 2, significant relationships were found between perceived physical competence and locomotor skills for boys only; whereas significant correlations were found between perceived physical competence and object control skills were found for both.

Because there were significant differences between the boys’ and girls’ motor skills and perceptions of physical competence, separate linear regression analyses were calculated to predict perceptions of physical competence. After controlling for age in months and BMI, both the regression model for locomotor skills *F*(3, 123) = 3.047, *p* = 0.005, *R*^2^ = 0.101 and for object control skills *F*(3, 123) = 4.243, *p* = 0.007, *R*^2^ = 0.097 predicted girls’ perceived competence in kindergarten. Similarly, both regression models for kindergarten boys were statistically significant: locomotor skills *F*(3, 121) = 3.047, *p* = 0.031, *R*^2^ = 0.071; and object control skills *F*(3, 121) = 4.887, *p* = 0.003, *R*^2^ = 0.110. In grade 2, neither of the regression models were statistically significant for girls: locomotor skills *F*(3, 123) = 0.393, *p* = 0.759, *R*^2^ = 0.011; and object control skills *F*(3, 123) = 1.361, *p* = 0.259, *R*^2^ = 0.036. For grade 2, however, locomotor skills *F*(3, 121) = 4.493, *p* = 0.005, *R*^2^ = 0.112 and object control skills *F*(3, 121) = 8.424, *p* < 0.001, *R*^2^ = 0.191 significantly predicted perceptions of physical competence.

To examine the generalizability of the findings of this study, we tested whether the 250 children in the longitudinal sample were representative of children not included in the longitudinal sample in both kindergarten (*n* = 137) and grade 2 (*n* = 143) from the same schools. Using independent *t*-tests, children’s locomotor skills, object control skills, and perceptions of physical competence scores were compared. No significant differences were found between the two groups for locomotor raw scores *t*(386) = −0.417, *p* = 0.677, object control raw scores *t*(386) = 0.405, *p* = 0.685, or perceptions of physical competence *t*(386) = −0.613, *p* = 0.541 in kindergarten. Similarly, no significant differences were found between the two groups for locomotor raw scores *t*(391) = 1.587, *p* = 0.113, object control raw scores *t*(391) = 1.650, *p* = 0.653, or perceptions of physical competence *t*(391) = 0.457, *p* = 0.459 in grade 2. These findings suggest that the longitudinal sample was representative of the larger cross-sectional samples.

## 4. Discussion

Examining the relationship between perceived and actual motor skill proficiency as children transition from early to middle childhood provides important developmental information about how perceptions are forming and when they begin to become more accurate. We tested aspects of a developmental model conceptualized by Stodden and colleagues [15]: that the relationship between motor skill proficiency and perceptions of physical competence would strengthen as children transitioned from early to middle childhood. Further, we extended this thought by including gender as a factor affecting these relationships.

Consistent with the principle that motor development is cumulative [34,35], the children’s locomotor and object control skills significantly improved from kindergarten to grade 2. The POMP scores showed that locomotor skills improved 7% for boys and 10% for girls, while object control skills improved by 15% and 14% for boys and girls, respectively. More detailed analyses of individual skills revealed that girls improved in all six locomotor skills and most object control skills, with the roll and throw being the exceptions (Table 2). The boys also significantly improved in all six object control skills and demonstrated significant improvements in three of the six locomotor skills. The boys’ gallop, leap, and jump did not change (Table 2). These findings are consistent with previous research showing that boys perform better with object control skills [10,12,17,18,26,27]; they also support studies showing that girls have more mature locomotor skills than boys of the same age [10,12,18]. The locomotor (gallop, leap, and jump) and object control skills (roll and throw) that did not improve in our study may reflect the types of activities children are engaging in and practicing. For example, both the boys and girls significantly improved their kicking and running, two predominant skills associated with soccer. In Canada, soccer is the most popular team sport for children and youth [36]. One in four respondents in a nationwide survey reported having at least one child living in the household playing soccer on a regular basis [37], and more than 750,000 children in Canada are part of an organized soccer program [36]. The impact that experience and/or practice can have on motor skill proficiency is evident in Williams and colleagues’ [38] study on throwing patterns of boys and girls. These authors found that young boys and girls did not differ in throwing velocity when using their non-dominant hand. However, boys threw significantly harder than girls when using their dominant hand, which demonstrated that the large differences in throwing when children use their dominant hand could be attributed to practice [38]. Opportunities to engage in activities and play often conform to stereotypical gender norms, reflecting sociocultural influences and beliefs about how boys and girls should act [34]. There is some evidence to suggest that girls in the school district in this study play fewer team sports and participate in more dance than boys [39]; there is also evidence from the broader literature indicating that girls may play more of a spectator role in territory invasion activities such as soccer [40]. These participation patterns likely have an effect on the specific motor skills that boys and girls develop, and as Stodden et al. [15] suggested, these motor skills are the tools that enable participation.

Perceptions of physical competence raw scores were high in kindergarten and in grade 2, which support previous findings reporting higher levels of perceived physical competence in early childhood [1,16,18,19] and middle childhood [19]. Our second hypothesis was that perceptions of physical competence would decrease from kindergarten to grade 2. Although the change in perceived physical competence was statistically significant from kindergarten to grade 2, the effect size (ηp2 = 0.02) was small [41], equating to an increase in perceived physical competence raw score of approximately one on a scale ranging from 6 to 24. Although the change was small, it was surprising that perceptions of physical competence increased, which is not consistent with developmental theory [13,14,15].

While it was expected that perceptions of physical competence would decrease from kindergarten to grade 2, we actually found that perceptions increased for boys and girls. Similarly, the children’s fundamental motor skills significantly increased from kindergarten to grade 2. This is one of the very few studies to examine children’s perceptions of physical competence during this transitional period of development. Spessato et al. [19] found a modest significant relationship between perceptions and motor skill proficiency among 6-year-old children, and a similar strength relationship that was not significant among 7-year-old children. However, their sample was not stratified by sex. Our findings show that the relationships between fundamental motor skills and perceived physical competence were significant for both boys and girls in kindergarten, and became stronger for boys by grade 2. The strengthened relationship for boys may reflect a positive internalization of their increased proficiency. For girls however, the relationship weakened as they aged.

The girls’ perceptions of physical competence increased, while the relationship with motor competence decreased. This suggests that factors other than the motor skills may be influencing the formation of girls’ perceptions of physical competence. It is possible that the TGMD-2 did not capture a specific range of motor skills that influence girls’ perceptions of competence. Previous studies have suggested that slightly older girls may discount their abilities and therefore have lower perceptions of physical competence [2,42]. However, this is not the case in this study, where girls’ perceptions of physical competence stayed high in grade 2 and were significantly higher than the boys. Data previously collected with grade 2 students in the same eight schools showed that girls participated in significantly more swimming, gymnastics, and formal and informal dance compared to boys [43]. The skills used for these types of activities are not well captured in the TGMD-2 and, therefore, the potential to see the relationship between motor skill proficiency and perceived physical competence is likely diminished.

A limitation of longitudinal research and the current study is the loss to follow-up through study withdrawal or other factors (e.g., switching schools). However, our comparisons of the cross-sectional and longitudinal samples did not reveal significant differences in any of the variables, which suggests that the longitudinal sample in this study was representative of the larger sample of kindergarten and grade 2 children. Another limitation of the study is that the survey used to assess perceived physical competence had few questions pertaining to specific object control skills (no questions in kindergarten and one question in grade 2). As a result, stronger relationships may have been established if the tool used in this study more closely reflected the motor skills tested. In the future, researchers may wish to also measure children’s perceptions of their abilities in activities they participate in. Finally, the physical activity levels of children were not measured in this study, which may have impacted the relationship between motor competence and perceived physical competence, and should be taken into consideration when interpreting the findings.

## 5. Conclusions

Children’s perceptions of their physical competence are important in the development of the self and ultimately can encourage or discourage children and youth’s participation in physical activities and sport [14,44]. Our findings suggest that the change in the relationship between fundamental motor skill proficiency and perceived physical competence as children transition from early to the beginning of middle childhood is statistically different for boys and girls. With that, factors other than motor skills may be influencing girls’ perceptions. Explanatory models may need to be altered to represent the influence of sex. Efforts need to be made to better understand what factors influence girls’ perceived physical competence and what that means for the girls’ cognitive, social, and physical development.

## Figures and Tables

**Table 1:** Raw scores for motor skills, perceived physical competence, and POMP score in kindergarten and grade 2.

Subscale Raw Scores (Range)		Kindergarten			Grade 2		
		Mean	SD	POMP	Mean	SD	POMP
Locomotor skills (0–48)	Boys	25.8	7.1	53.9	29.3	5.6	61.0
	Girls	26.9	6.8	56.2	31.9	4.9	66.6
Object control skills (0–48)	Boys	23.6	8.0	49.2	31.5	6.3	65.6
	Girls	19.8	6.5	41.3	27.2	5.8	56.8
Perceived physical competence (6–24)	Boys	18.2	3.2	76.0	19.5	3.1	81.4
	Girls	19.3	2.7	80.4	20.5	2.5	85.5

Note. POMP = Percent of maximum possible score (range 0–100).

**Table 2:** Examining the differences of individual skills from the TGMD-2 using paired-sample *t*-test.

Skill	Boys (*n* = 124)					Girls (*n* = 126)				
	Kindergarten		Grade 2		*p*	Kindergarten		Grade 2		*p*
	M	SD	M	SD		M	SD	M	SD	
Run	5.6	1.8	6.6	1.5	<0.001 **	5.3	1.9	6.7	1.2	<0.001 **
Gallop	3.6	2.2	4.1	1.9	0.060	4.2	2.1	4.9	1.6	0.004 *
Hop	4.7	1.9	5.5	1.7	<0.001 **	5.0	1.9	5.9	1.8	<0.001 **
Leap	3.2	1.3	3.2	1.1	0.912	2.8	1.9	4.0	1.6	<0.001 **
Jump	3.5	2.2	3.6	2.2	0.629	3.3	2.1	4.1	2.0	<0.001 **
Slide	5.0	2.4	6.0	1.3	<0.001 **	5.3	2.3	6.1	1.3	<0.001 **
Strike	6.4	2.0	7.1	1.6	<0.001 **	5.2	2.0	5.9	1.7	0.006 *
Dribble	2.4	2.3	5.5	2.1	<0.001 **	2.2	2.1	4.6	1.9	<0.001 **
Catch	3.2	1.5	4.0	1.4	<0.001 **	3.1	1.5	4.2	1.4	<0.001 **
Kick	5.1	1.6	6.2	1.1	<0.001 **	4.4	1.6	5.6	1.0	<0.001 **
Throw	3.1	2.4	4.5	2.1	<0.001 **	1.8	1.3	1.9	1.3	0.895
Roll	3.1	2.0	4.1	1.6	<0.001 **	3.7	1.3	3.9	1.1	0.238

Note. * *p* < 0.05, ** *p* < 0.001

**Table 3:** The relationship between motor skills and perceived physical competence.

TGMD-2 Subtest	Perceived Physical Competence			
	Kindergarten		Grade 2	
	Boys	Girls	Boys	Girls
Object control skills	0.326 *	0.288 **	0.450 **	0.193 *
Locomotor skills	0.271 **	0.255 **	0.327 **	0.064

Note. * *p* < 0.05 and ** *p* < 0.001.
